# Stakeholder efforts to mitigate antiretroviral therapy interruption among people living with HIV during the COVID‐19 pandemic in China: a qualitative study

**DOI:** 10.1002/jia2.25781

**Published:** 2021-09-02

**Authors:** Yinghui Sun, Yuewei Zhan, Hui Li, Tanwei Yuan, Yanxiao Gao, Bowen Liang, Anping Feng, Peiyang Li, Weiran Zheng, Thomas Fitzpatrick, Dan Wu, Xinyi Zhai, Huachun Zou

**Affiliations:** ^1^ School of Public Health (Shenzhen) Sun Yat‐sen University Shenzhen China; ^2^ Shizhong District Center for Disease Control and Prevention Jinan China; ^3^ Department of Internal Medicine University of Washington Seattle Washington USA; ^4^ London School of Hygiene & Tropical Medicine London UK; ^5^ Danlan Goodness BlueCity Holdings Ltd. Beijing China; ^6^ School of Public Health Shanghai Jiao Tong University School of Medicine Shanghai China; ^7^ Kirby Institute University of New South Wales Sydney New South Wales Australia

**Keywords:** antiretroviral therapy, antiretroviral therapy maintenance, community‐based organization, COVID‐19, HIV, people living with HIV

## Abstract

**Introduction:**

The COVID‐19 pandemic has affected antiretroviral therapy (ART) continuity among people living with HIV (PLHIV) worldwide. We conducted a qualitative study to explore barriers to ART maintenance and solutions to ART interruption when stringent COVID‐19 control measures were implemented in China, from the perspective of PLHIV and relevant key stakeholders.

**Methods:**

Between 11 February and 15 February 2020, we interviewed PLHIV, community‐based organization (CBO) workers, staff from centres for disease control and prevention (CDC) at various levels whose work is relevant to HIV care (CDC staff), HIV doctors and nurses and drug vendors from various regions in China. Semi‐structured interviews were conducted using a messaging and social media app. Challenges and responses relevant to ART continuity during the COVID‐19 pandemic were discussed. Themes were identified by transcript coding and mindmaps.

**Results:**

Sixty‐four stakeholders were recruited, including 16 PLHIV, 17 CBO workers, 15 CDC staff, 14 HIV doctors and nurses and two drug vendors. Many CDC staff, HIV doctors and nurses responsible for ART delivery and HIV care were shifted to COVID‐19 response efforts. Barriers to ART maintenance were (a) travel restrictions, (b) inadequate communication and bureaucratic obstacles, (c) shortage in personnel, (d) privacy concerns, and (e) insufficient ART reserve. CBO helped PLHIV maintain access to ART through five solutions identified from thematic analysis: (a) coordination to refill ART from local CDC clinics or hospitals, (b) delivery of ART by mail, (c) privacy protection measures, (d) mental health counselling, and (e) providing connections to alternative sources of ART. Drug vendors contributed to ART maintenance by selling out‐of‐pocket ART.

**Conclusions:**

Social and institutional disruption from COVID‐19 contributed to increased risk of ART interruption among PLHIV in China. Collaboration among key stakeholders was needed to maintain access to ART, with CBO playing an important role. Other countries facing ART interruption during current or future public health emergencies may learn from the solutions employed in China.

## 1 INTRODUCTION

Access and adherence to antiretroviral therapy (ART) is needed for people living with HIV (PLHIV) to achieve viral suppression, reduce HIV‐associated morbidity and mortality, and prevent HIV transmission [1, 2]. Over the last two decades, China has made large strides in expanding access to ART. In 2003 China adopted the ‘Four‐Free One‐Care’ policy, under which all PLHIV in the country are able to access free ART from designated healthcare facilities, including hospitals specialized in infectious diseases and clinics affiliated to centres for disease control and prevention (CDC) at various levels [3]. Thanks to this policy, the coverage of free ART among diagnosed PLHIV in China increased from 64.2% in 2006 to 86.6% in 2019 [4]. PLHIV who do not receive government‐subsidized ART often purchase medications out‐of‐pocket at pharmacies or from drug vendors via the internet [5].

HIV‐themed community‐based organizations (CBO) in China provide health education, counselling, advocacy and treatment monitoring services for PLHIV and people at risk of HIV infection, including men who have sex with men (MSM), sex workers, and people who inject drugs. From 1988 to 2009, the number of CBO working in the field of HIV prevention and care in China increased from zero to over 400, with one‐third and one‐fourth of these organizations focusing on the needs of MSM and PLHIV, respectively [6]. The number of CBO dedicated to HIV prevention and care in China exceeded 700 in 2019 [7]. CBO play an important role in promoting and connecting PLHIV and at‐risk groups to free health services, including HIV testing and ART [8].

To control the spread of COVID‐19, China introduced a series of public health responses. On 23 January 2020, a citywide lockdown was imposed in Wuhan. On 26 January 2020, COVID‐19 control measures were imposed nationwide [9]. Travel restrictions and suspension of postal services were implemented across all 31 provinces and administrative regions in mainland China. Citywide lockdowns and enforced home quarantine were implemented in areas with confirmed COVID‐19 cases.

While China achieved significant success in containing COVID‐19, these measures also impeded the HIV care services. To maintain access to ART, the National Center for AIDS/STD Control and Prevention (NCAIDS) published updated policy guidelines on 26 January 2020, which allowed PLHIV to refill one‐month of ART from any designated hospital or clinic [10]. Despite these efforts, more than one‐third of PLHIV in China reported risk of ART interruption at the early stage of the COVID‐19 outbreak [11]. PLHIV residing in areas that implemented citywide lockdowns and travel restrictions were at higher risk of ART interruption. Themes and theory generated in qualitative study can explain and predict various outcomes within diverse contexts of the healthcare system [12, 13]. However, to date, there is still a lack of qualitative information on ART maintenance in the context of COVID‐19. To better characterize barriers to ART maintenance and solutions to ART interruption in China during the implementation of stringent COVID‐19 control measures, we conducted a qualitative study among PLHIV and relevant key stakeholders.

## 2 METHODS

### 2.1 Study design and participants

Between 11 February and 15 February 2020, we interviewed PLHIV and representatives from the following stakeholder groups: CBO workers, CDC staff, HIV doctors and nurses, and drug vendors [3, 5, 6]. Interviews were semi‐structured and designed based on discussions with a panel of eight experts in the fields of ART delivery and HIV epidemiology. Participants were recruited from all seven geographic regions of China, including northern China (e.g., Beijing, Tianjin), eastern China (e.g., Shanghai, Hefei), central China (e.g., Wuhan, Zhengzhou), southern China (e.g., Shenzhen, Nanning), northwestern China (e.g., Urumqi, Tianshui), southwestern China (e.g., Chongqing, Chengdu) and northeastern China (e.g., Shenyang, Harbin). Participants were selected by three HIV clinicians, one HIV social worker and four HIV epidemiologists using purposive sampling. Eligible participants were invited to complete an interview via WeChat or telephone. Eligible PLHIV were at least 18 years old and currently on ART. Eligible CBO workers, CDC staff, HIV doctors and nurses, as well as drug vendors were at least 18 years old and held a position related to ART delivery. Study protocol is shown in the Supporting Materials in Additional file 1.

### 2.2 Data collection

Each interview was conducted online via WeChat, a multipurpose messaging and social media application (Tencent, Shenzhen). Participants were allowed to be interviewed via either a voice call or text messaging. Each interview was one‐on‐one and lasted for approximately 20 minutes. Demographic characteristics, including age, gender and geographic location, were collected. PLHIV were asked about ART history, barriers to obtaining ART in the midst of stringent COVID‐19 control measures and solutions to solve ART interruption. For this study, the period of stringent COVID‐19 control measures was defined as 10 January 2020, when the Chinese New Year holiday began and 15 February 2020, when our study ended. Many PLHIV left the city where they typically accessed HIV care during this period and were subsequently affected by travel restrictions and home quarantine requirements. CBO workers, CDC staff, HIV doctors and nurses, as well as drug vendors were asked to describe their work conditions related to ART delivery, barriers to ART delivery and solutions to maintain HIV care during this period. Semi‐structured interview guides are available in Tables Figure S1‐S5 in Additional file 1 (Supporting Materials). Data collection was stopped after thematic saturation was reached (i.e., when new data produced little or no new information to address the study question).

### 2.3 Data analysis

**Figure 1:**
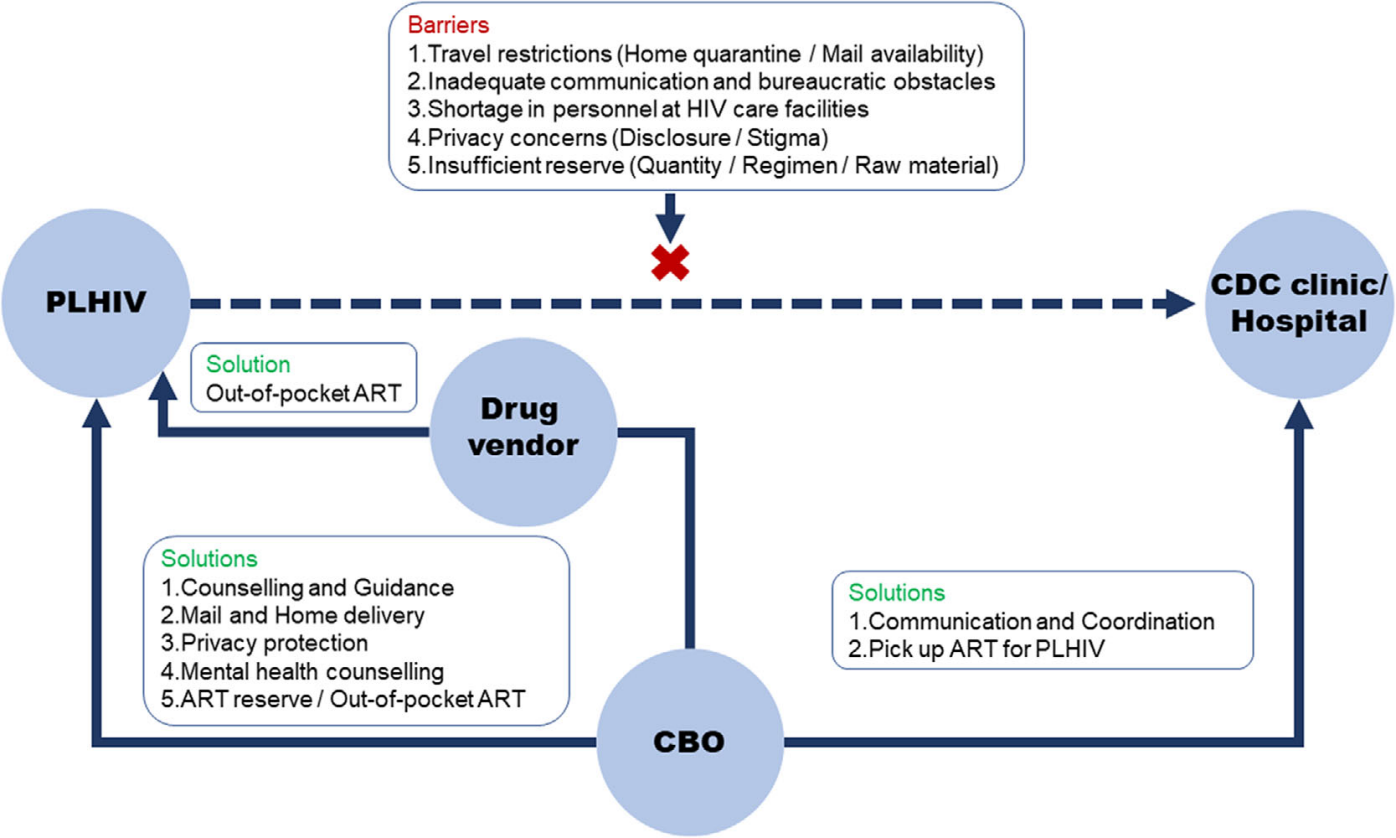
Joint efforts to mitigate ART interruption by CBO, PLHIV, CDC clinics, hospitals and drug vendors. ART, antiretroviral therapy; CBO, community‐based organizations; CDC, centres for disease control and prevention; PLHIV, people living with HIV.

Voice calls were audio recorded, transcribed in Mandarin and then encoded using NVivo 11.0 (QSR International, Melbourne, Australia). Thematic analysis was used to analyse and explore potential themes. We followed an analytical process recommended by previous studies [14, 15, 16]: (a) familiarizing yourself with your data, (b) generating initial codes, (c) searching for themes, (d) reviewing themes, (e) defining and naming themes, and (f) producing the report. We formed detailed codebooks for each of the five stakeholder groups. An inductive approach was used to develop data‐driven themes. We applied two prespecified themes: (a) barriers to ART maintenance, and (b) solutions to ART interruption. We used a mindmap (see Figure S1 in Additional file 2) [17] to summarize our prespecified themes before beginning thematic analysis. After discussion, we found CBO participated in all identified aspects of barriers to ART maintenance and solutions to ART interruption. Consequently, we refined and renamed themes from the perspective of CBO. Consensus on identified themes was reached after discussion. Descriptive statistics were used to summarize demographic characteristics. Informative quotations from participants were translated to English and checked for accuracy. A relationship framework among different stakeholders was generated to visually summarize our results (Figure 1) [15].

After conducting inductive analysis in a data‐driven way, we performed higher order analysis based on the findings, aiming to raise the generated themes to a conceptually theoretical level. We applied the theoretical framework based on the Andersen's Behavioral Model of Health Service Use (ABM) [18], which is available to understand how patient and environmental factors impact health behaviours and outcomes, and has been applied in other qualitative studies about barriers and facilitators of health programmes [19]. We combined our results presented in Figure 1 with the ABM, to further explore how patients and environmental factors affect ART maintenance.

### 2.4 Ethical statement

This study was approved by the Sun Yat‐sen University Ethics Committee (SYSU‐SPH2020008). Informed consent was obtained from each participant prior to interview.

## 3 RESULTS

### 3.1 Characteristics of participants

Of 69 individual stakeholders invited, 64 (92.8%) completed an interview, including 16 PLHIV, 17 CBO workers, 15 CDC staff, 14 HIV doctors and nurses and two drug vendors. Median age of PLHIV was 29 years (interquartile range (IQR): 24 to 37 years) and median duration since ART initiation was 2.5 years (IQR: 0.5 to 3.5 years). All CBO workers were men, more than 50% of whom had worked in HIV care services for over 10 years (IQR: six to twelve years) (Table 1). Among the 17 CBO workers we interviewed, 11 CBO provided services to PLHIV exclusively and six provided services to both PLHIV and MSM.

**Table 1:** Characteristics of participants

	PLHIV (*n* = 16)	CBO workers (*n* = 17)	HIV doctors and nurses (*n* = 14)	CDC staff (*n* = 15)	Drug vendors (*n* = 2)
Gender					
Men	14 (87.5%)	17 (100.0%)	3 (21.4%)	8 (53.3%)	2 (100.0%)
Women	2 (12.5%)	0 (0.0%)	11 (75.6%)	7 (46.7%)	0 (0.0%)
Age (years), (median, IQR)	29.0 (24.0 to 37.0)	38.0 (32.0 to 45.0)	41.0 (35.0 to 49.0)	39.0 (34.5 to 48.5)	‐
Years on ART (median, IQR)	2.5 (0.5 to 3.5)^a^	‐	‐	‐	‐
Years at work (median, IQR)	‐	10.0 (6.0 to 12.0)^b^	9.5 (4.0 to 12.5)	10.5 (5.3 to 15.0)	‐
Geographical distribution					
Northern China	1 (6.3%)	3 (17.6%)	1 (7.1%)	2 (13.3%)	1 (50.0%)
Eastern China	5 (31.3%)	4 (23.5%)	2 (14.3%)	5 (33.3%)	1 (50.0%)
Central China	8 (50.0%)	3 (17.6%)	0 (0.0%)	2 (13.3%)	0 (0.0%)
Southern China	1 (6.3%)	3 (17.6%)	4 (28.6%)	2 (13.3%)	0 (0.0%)
Northwestern China	1 (6.3%)	2 (11.8%)	0 (0.0%)	3 (20.0%)	0 (0.0%)
Southwestern China	0 (0.0%)	0 (0.0%)	3 (21.4%)	1 (6.7%)	0 (0.0%)
Northeastern China	0 (0.0%)	2 (11.8%)	4 (28.6%)	0 (0.0%)	0 (0.0%)

Abbreviations: ART, antiretroviral therapy; CBO, community‐based organization; CDC, centres for disease control and prevention; IQR, interquartile range; PLHIV, people living with HIV.

^a^ One missing

^b^ One missing.

### 3.2 Barriers to ART maintenance

Despite NCAIDS policy guidelines designed to facilitate ART maintenance during the COVID‐19 pandemic, travel restrictions continued to have an impact on obtaining ART. Refilling ART prescriptions was also impeded by inadequate communication between PLHIV and HIV care facilities as well as cumbersome bureaucratic processes. CDC staff, HIV doctors and nurses struggled to balance their time between ART delivery and COVID‐19 response efforts. Some HIV doctors and nurses were redeployed to respond to COVID‐19 full‐time, leaving no time for ART delivery. For PLHIV, privacy and unwanted disclosure of HIV status was a salient concern when obtaining ART, particularly among those who were unexpectedly forced to home quarantine with family members. Insufficient ART reserve in CDC clinics and hospitals, and among drug vendors, was a barrier to ART maintenance. Detailed quotations are shown in Table 2.

**Table 2:** Barriers to ART maintenance from perspectives of different stakeholders

**Barriers**	**Quotations**
Travel restrictions	‘Some (PLHIV) live in the countryside. They cannot leave and courier services cannot reach them, so there is no solution’. (CBO, No.10, 40‐year‐old man, Zhengzhou) ‘Because the SF courier company would only deliver to towns and could not go into the countryside because of road closures, a lot of rural PLHIV had to sneak out of the countryside, walking for 40 kilometres, groping blindly in the dark [to get ART]!’ (drug vendor, No. 2, man, Beijing)
Inadequate communication and bureaucratic obstacles when attempting to refill ART	‘A [staff member at a local health facility] told me to ask the local CDC clinic for help. Later, he told me to contact the AIDS prevention office, and then the health bureau. After all that running back and forth I was still unable to solve my ART problem’. (PLHIV, No. 6, 42‐year‐old woman, Baise)
	‘The local CDC clinic has a lot of requirements, such as a letter from one's designated CDC clinic or hospital. It was difficult for people to track down the chief of a CDC clinic or hospital [to obtain a letter], so most [PLHIV] chose not to do this’. (CDC, No. 5, 36‐year‐old man, Guangzhou)
Shortage in personnel at HIV care facilities	‘[PLHIV] could not get in touch with local doctors responsible for ART delivery because most doctors were mobilized to the front lines to fight the pneumonia outbreak’. (CDC, No. 7, 46‐year‐old man, Xiaogan)
Privacy concerns of PLHIV	‘A particularly serious problem was that [PLHIV] who typically work outside their hometowns had returned home to visit family members for Spring Festival. They had to conceal from their family that they were taking ART, and also hide their medications where no one would find them. They also worried whether regular daily contact would infect family members. These problems created huge amounts of stress for PLHIV, and some felt they were going to go crazy. They could not leave due to home quarantine, but if they stayed their family would probably discover they are infected, so it was incredibly stressful’. (CBO, No. 12, 54‐year‐old man, Cangzhou)
Insufficient reserve in CDC clinics and hospitals and among drug vendors	‘Recently, the ART supply chain has been seriously disrupted, especially medications in their original packaging, like TRIUMEQ and DTG (combination of dolutegravir, abacavir and lamivudine), which are now out of stock across the country, no one can buy them, because these medications do not come from India, but from South Africa. In the past medications from South Africa were brought back by migrant workers returning home to China, but this avenue is now totally cut‐off’. (drug vendor, No. 2, man, Beijing)

Abbreviations: ART, antiretroviral therapy; CBO, community‐based organization; CDC, centres for disease control and prevention; PLHIV, people living with HIV. In the quotations, ‘(...)’ represented the explanation of the former word/phrase in the original sentence, and ‘[...]’ represented the contextual supplement to the missing sentence elements in the original sentence.

### 3.3 CBO solutions to ART interruption

CBO played an important role in ART maintenance during the COVID‐19 pandemic. Specifically, CBO provided PLHIV with guidance and coordination to refill ART, mail and home delivery services, assistance in privacy protection, mental health counselling and connections to alternative sources of ART. Furthermore, CBO assisted CDC clinics and hospitals by coordinating ART delivery to PLHIV.

#### 3.3.1 Coordination to refill ART from local CDC clinics or hospitals

While the NCAIDS directive allowed PLHIV to refill ART from any HIV care facility in China, the specific process was initially unclear to both PLHIV and healthcare providers. CBO played an intermediary role by guiding PLHIV in their attempts to refill ART. They referred PLHIV to online guidelines, helped PLHIV contact local CDC clinics and hospitals and shared experiences with other CBO.

I knew about the [NCAIDS policy], so I contacted my local CDC clinic and infectious diseases hospital that manages ART and asked for details about the policy, but honestly speaking they were not clear on the policy themselves and could only point to the policy document itself. So, we disseminated the specifics on policies [to obtain ART during the COVID‐19 pandemic] through our WeChat account, and bit by bit some PLHIV successfully refilled ART from local CDC clinics and treatment centres using these policies. Then we collected their successful experiences and immediately shared them with the rest of the service community. (CBO, No. 2, 35‐year‐old man, Hefei)

For PLHIV who did not know how to contact CDC clinics or hospitals, CBO contributed by finding and providing contact information.

[PLHIV] needed their designated hospital to provide a work letter, but many PLHIV do not know the phone number of their designated hospital. I found the number for them, then they could contact their designated hospitals, and a nurse would provide them with the needed documentation. (CBO, No. 13, 56‐year‐old man, Tianjin)

#### 3.3.2 Delivery of ART by mail

In‐person ART collection was limited by travel restrictions during the COVID‐19 pandemic, so mail delivery became a major source for ART refills for PLHIV in China. Because CDC staff, HIV doctors and nurses were overwhelmed by COVID‐19 work, CBO workers became responsible for obtaining ART and mailing it to PLHIV.

Some hospitals did not offer ART delivery through mail. When PLHIV provided their information, we could go to the hospital to collect their ART, and then assisted the hospital in mailing ART to them. (CBO, No. 7, 38‐year‐old man, Nanning)

Although public transportation was closed or suspended in many locations, hospitals still tried to deliver ART to PLHIV with the help of CBO.

[We] tried our best to send [ART] to PLHIV through CBO. For example, if ground mail wasn't possible, we'd try air mail, or various courier companies, even if only one method worked, we would still strive to send out [ART]. (doctor, No. 8, 39‐year‐old man, Shenyang)

Because of travel restrictions and home quarantine, PLHIV were unable to leave their towns or neighbourhoods to collect ART. As a solution, some CBO workers physically delivered ART to PLHIV at their homes.

I am also a CBO worker, so I mobilized some of our volunteers. When courier services could not reach a certain area, I would have CBO volunteers contact PLHIV, and if the volunteers happened to live near a PLHIV, they would drive over to the village pretending they were dispensing food and deliver some ART, making sure PLHIV could get ART in time. (doctor, No. 13, 48‐year‐old man, Tieling)

Home delivery was an effective way to achieve ART maintenance, especially in places with strict citywide lockdowns or home quarantines.

In Wuhan, there were volunteers and cars with travel passes who would pick up [ART] and deliver it to villages and neighbourhoods, then [PLHIV] could come to get ART themselves. (CBO, No. 16, 33‐year‐old man, Shenzhen and Xiamen)

#### 3.3.3 Privacy protection measures

Privacy was a major concern surrounding ART delivery. PLHIV in China often do not disclose their HIV status to family members. Due to home quarantine, many PLHIV were confined at home with family members who did not know their HIV status. Stakeholders, including CBO workers, tried to protect the privacy of PLHIV by removing labelling from ART, mailing medications anonymously and mailing from home rather than workplace addresses.

Most [PLHIV] asked us to remove all packaging from the [ART], including labels. We are quite experienced at this since clients have often asked us to help remove medication labelling in the past. In this way even if family members find a bottle of medication, there is nothing for them to read. Some asked us to write a nickname rather than their real name on the delivery packaging because they were very worried that a neighbour might see their name and say something to their family members. Others asked us to specify on the delivery packaging that the included item was a ‘daily necessity’ rather than medication. (CBO, No. 15, 38‐year‐old man, Qingdao)

Mailing ART from a worker's home address rather than the CBO's workplace address helped PLHIV protect their HIV status from unwanted disclosure.

We did not mail ART from our workplace, because it is inappropriate to write the name of our organization on the delivery packaging. Instead, we mailed it from our home address. (CBO, No. 1, 31‐year‐old man, Shenzhen)

#### 3.3.4 Mental health counselling

At the time interviews were conducted, PLHIV had been quarantined at home for more than half a month. In addition to fears about ART interruption, many also worried their HIV status may place them at higher risk of critical illness from COVID‐19. PLHIV frequently mentioned secretly taking ART at home and fears of inadvertently revealing their HIV status to family members were sources of mental stress. To respond to increasing rates of anxiety, depression and insomnia among PLHIV, CBO provided mental health counselling to PLHIV through smartphone‐based instant messaging applications.

Some (PLHIV) were anxious and irritable. We would use periods of free time, like after 8 PM or 9 PM, to chat with them via WeChat or QQ, and in the process provide some mental health counselling. (CBO, No. 12, 54‐year‐old man, Cangzhou)

Some PLHIV did not seek mental health counselling even if they had underlying mental illness. CBO took the initiative to support this vulnerable group.

Of course, we would also actively find PLHIV who were prone to depression. I would ask how they were doing at home and give them some recommendations, such as reading, listening to music and exercise, and through these activities relieve the mental pressure of home quarantine. (CBO, No. 15, 38‐year‐old man, Qingdao)

Although CBO could not guarantee a professional level of mental health counselling, the emotional support of CBO workers was important to PLHIV.

We could not provide professional counselling because we are not professional counsellors or psychologists. But we could still give general relief and comfort. (CBO, No. 2, 35‐year‐old man, Hefei)

PLHIV reported CBO not only helped them by coordinating with CDC clinics and hospitals, but also encouraged them to persevere in efforts to obtain ART.

I experienced ART interruption for six days and couldn't get any sleep. So I joined a chat group for PLHIV who had experienced or were at risk of ART interruption due to the pandemic through an official account and described my situation. A CBO worker added my WeChat account to call me and ask about my wellbeing. She also gave me the phone number to the director of a local CDC clinic and explained my situation to the director. The CDC clinic said I could borrow medicine [from a local hospital] but could not arrange for delivery of medicine. The CBO worker asked me about my situation every day and was more considerate than my doctor. Every time I was about to give up, I remembered how concerned the CBO was about me, and insisted on getting my medicine. Finally, the CBO worker contacted the pandemic response headquarters, and they were willing to help me by contacting the local CDC director and had them deliver medicine to me. Although I had been off ART for nine days, after so many days of hard work, I finally got my ART. The CBO worker and I were very happy. When I told my friends the story, they said it was incredible. I think this is the power of persistence. I really want to thank the CBO. I also want to thank the pandemic response headquarters staff who answered the phone, the office director and the person who drove me to get the medicine. Thank you all. (PLHIV, No. 5, 29‐year‐old man, Xinjiang)

#### 3.3.5 Providing connections to alternative sources of ART

While CBO are typically not responsible for ART delivery in China, some do have small reserves of ART. During the COVID‐19 pandemic, these ART reserves were used to address ART interruption. ART reserves were typically collected through donations from PLHIV or larger CBO.

ART reserves at CBO had been collected in normal times. For instance, when patients changed their ART prescription, they may have a half bottle of ART left, which would be donated for others to use in an emergency. CBO gradually accumulated ART over time, which were all free medications. Other CBO might also have extra ART that they could donate to us. (CBO, No. 12, 54‐year‐old man, Cangzhou)

As the pandemic continued, CBO ran out of free ART to distribute. Buying ART out‐of‐pocket became an option for some PLHIV to avoid ART interruption.

We also suggested some PLHIV purchase ART out‐of‐pocket for a short period if they could not get free ART. We would help them pick up ART after purchasing it and mail it to them. But in the end this solution was only appropriate for a small number of people because most still need free medication. (CBO, No. 2, 35‐year‐old man, Hefei)

### 3.4 Other solutions

Drug vendors also mitigated the risk of ART interruption by selling ART to PLHIV who could afford out‐of‐pocket ART.

ART like Kocitaf (combination of dolutegravir, emtricitabine and tenofovir) have few side effects and are well tolerated, so it is relatively safe to replace [free ART] temporarily. (drug vendor, No. 2, man, Beijing)

Overall, stakeholders made significant contributions to efforts to avoid interruption in ART during the COVID‐19 pandemic. CBO acted as a bridge between PLHIV, CDC clinics and hospitals. Figure 1 depicts the relationship among stakeholders. Factors of patient and environment, both contributed to barriers to ART maintenance and solutions to ART interruption. And detailed analysis and description grouped by ABM domains are given in Table 3.

**Table 3:** Barriers to ART maintenance and solutions to ART interruption, categorized by ABM domains

	**Barriers**	**Solutions**
Patient characteristics		
Predisposing factors	Stigma Mental problem Potential ART interruption risk	Privacy protection Mental health counselling
Enabling factors	Travel restrictions (home quarantine) Privacy concern of HIV status disclosure	ART mail and home delivery Privacy protection
Perceived need	Concerns of ART interruption	Multiple stakeholder efforts to maintain ART delivery
Healthcare environment		
System factors	Shortage in personnel at HIV care facilities Insufficient reserve (quantity/regimen/raw material)	ART reserve in CBO Out‐of‐pocket ART recommended by CBO and bought from drug vendors
Clinic factors	Bureaucratic obstacles	Communication and coordination between PLHIV and CDC clinics/hospitals (CBO) Counselling and guidance
Provider factors	Inadequate communication	Communication and coordination between PLHIV and CDC clinics/hospitals (CBO) Counselling and guidance
External environment		
External factors	Travel restrictions (mail availability)	Pick up ART for PLHIV from CDC clinics/hospitals (CBO) ART mail and home delivery

Abbreviations: ABM, Andersen's Behavioral Model; ART, antiretroviral therapy; CBO, community‐based organization; CDC, centres for disease control and prevention; PLHIV, people living with HIV.

## 4 DISCUSSION

This is one of the first qualitative studies of multiple stakeholders to describe barriers to ART maintenance and solutions to ART interruption during the COVID‐19 pandemic in China. Travel restrictions, inadequate communication and bureaucratic obstacles between stakeholders, shortage in personnel, privacy concerns and insufficient ART reserve were barriers to ART maintenance. CBO took on an important role in responding to these barriers, acting as a bridge between PLHIV and ART providers. CBO workers helped PLHIV navigate bureaucratic requirements to refill ART from local CDC clinics and hospitals. They also took on new responsibilities during the COVID‐19 pandemic, delivering ART to PLHIV through the mail or in person, helping PLHIV protect their HIV status from unwanted disclosure and providing mental health counselling through social media applications.

We found that travel restrictions and home quarantine measures made it difficult for some PLHIV in China to refill ART from the designated CDC clinics or hospitals where they typically receive HIV care. This finding is consistent with large‐scale nationwide surveys of PLHIV in China where risk of ART interruption was correlated with travel away from home and living in areas that implemented citywide lockdowns during the COVID‐19 pandemic [11]. Despite nationwide policy reform efforts by the Chinese central government, PLHIV found local CDC clinics and hospitals to be slow and inconsistent in operationalizing policies designed to minimize the impact of COVID‐19 control measures on HIV care. CBO adopted new roles and novel measures to address these barriers. CBO workers learned how to refill ART through any local CDC clinic or hospital and then guided PLHIV through bureaucratic obstacles. Peer navigation has been identified as an important tool to optimize the HIV care continuum [20] and became particularly important during the COVID‐19 pandemic. CBO coordination of ART mail and home delivery services also helped PLHIV in China avoid ART interruption when faced with restrictions on mobility. Similar strategies have been employed in other low‐ and middle‐income countries (LMICs) responding to disruptions in HIV care from COVID‐19, including the Philippines and Pakistan [21, 22, 23]. Prior to the COVID‐19 pandemic, home delivery of ART by CBO was shown to improve early ART adherence and outcomes [24], and during the COVID‐19 pandemic this strategy has been identified as key to maintaining ART access in remote rural locations without reliable access to postal services [23, 25, 26]. Many regions including America, Europe, Asia and Africa benefited from CBO contributions to ART delivery during the COVID‐19 pandemic [25, 27, 28, 29, 30]. The role of CBO in ART provision could be similarly expanded in other low‐ and middle‐income countries to optimize ART maintenance. Efforts to maintain ART should take into consideration variation and complexity among different settings and apply suitable solutions in the country.

HIV‐related stigma and fear of disclosure of HIV status were common themes identified in our interviews of PLHIV in China. Stigma has been identified as a significant barrier to ART adherence in multiple contexts, and recent research has shown home quarantine during the COVID‐19 pandemic can exacerbate its influence [1, 31]. Many PLHIV in this study were reluctant to take ART while quarantined at home or apply for legal authorization to leave home to refill ART for fear that family members, partners, friends or neighbours would discover their HIV status. Delivery of ART by mail was preferred by some PLHIV because it allowed for refills to be obtained discretely. However, unwanted disclosure of HIV status to household members through labelling on packaging remained a concern identified in our interviews and in previous research of PLHIV in China [31]. CBO workers addressed this concern by removing identifying labels from both packaging and medication bottles before delivering ART to PLHIV in the mail or in person. CBO also provided a platform for PLHIV in China to voice their anxiety about privacy during home quarantine and solicit support from peers. Previous studies in other settings have highlighted the importance of addressing stigma among PLHIV through listening, counselling and communication when providing HIV care and ART [1, 32, 33]. Our findings also reflect challenges of stigma in maintaining ART and underscore the importance of sustained efforts to destigmatize HIV status and address PLHIV marginalization worldwide, both during and after the COVID‐19 pandemic. Additionally, expediencies such as removing ART label, anonymous mailing could help in privacy protection in constrained settings of social inclusiveness, and increase the ART adherence.

PLHIV in China reported increased anxiety, depression and insomnia during the COVID‐19 pandemic. Concerns regarding increased risk of critical illness from COVID‐19 infection and difficulty in obtaining ART were commonly reported sources of emotional stress in our interviews. Previous research found disruption caused by COVID‐19 has disproportionately affected the mental health of PLHIV in China as well as internationally [30, 31, 34]. CBO have historically provided mental health support to PLHIV in China, and these efforts were strengthened during the COVID‐19 pandemic. CBO workers provided counselling services outside of normal business hours, contacting clients at times when they could maintain privacy despite being quarantined at home with family members. Social media platforms and mobile apps were used to deliver counselling services to overcome barriers imposed by travel restrictions and citywide lockdowns. Studies in some high‐income countries and LMICs have found emotional support from CBO workers can help develop positive attitudes and beliefs among PLHIV, contributing to improved ART adherence [29, 35, 36, 37, 38]. Our results highlight that mental health counselling could be one effective option to mitigate mental stress and increase confidence to treatment among vulnerable groups like PLHIV in countries with strict travel restrictions.

Our study has several limitations. First, participants were recruited through purposive sampling. China is a large country of 1.5 billion people with significant regional diversity in terms of socio‐economic conditions and HIV care services. Consequently, our interviews may not represent the experiences of PLHIV or stakeholders in the whole country. To maximize the representativeness of our sample, we recruited participants from seven distinct geographical regions in China as well as from urban and rural settings. Second, we focused on the views of multiple stakeholders, while the actual feelings from PLHIV may be more practicable. Third, the flexibility inherent in our inductive approach to generating themes may lead to inconsistency and lack of coherence in identifying themes [14]. We closely followed a six‐step procedure in our thematic analysis to maximize consistency and reproducibility in our results [39]. Fourth, interviews were conducted during the early phase of the COVID‐19 outbreak in February 2020. Barriers to ART adherence and stakeholder responses may have changed as the pandemic continued to evolve, and these changes would not have been captured in this study. Finally, significant differences exist between PLHIV and delivery of HIV care across countries and regions. The barriers and solutions identified through our interviews may not be relevant or applicable to other contexts.

## 5 CONCLUSIONS

We found CBO played an important role in ART maintenance during the COVID‐19 pandemic in China. Innovative solutions implemented by Chinese CBO, including peer navigation to refill ART and CBO‐coordinated provision of ART through mail or home delivery services, could be useful in other regions of China as well as internationally. Expansion of CBO services faces deep structural limitations in China, which has prevented nongovernmental organizations in China from taking on a larger role in providing HIV care [40]. Healthcare policymakers should recognize the important role CBO have played in ensuring access to ART, particularly during the COVID‐19 pandemic, and further incorporate CBO into the existing ART delivery mechanism. Further research is needed to evaluate the long‐term cost‐effectiveness and feasibility of continuing CBO solutions to ART interruption after the COVID‐19 pandemic has ended. Efforts to maintain ART access during future public health emergencies should leverage collaborations between stakeholders, with CBO playing a crucial bridging role between government agencies and PLHIV.

## COMPETING INTERESTS

The authors declare that they have no competing interests.

## AUTHORS’ CONTRIBUTIONS

H.Z. conceived and designed the study in consultation with Y.S. and the other authors. H.Z. and Y.S. designed the questionnaire in consultation with H.L., Y.Z., B.L. and A.F. Y.S., Y.Z., B.L. and A.F. conducted the interview and obtained data, and, together with H.Z., D.W. and X.Z. conceived the analysis and presentation. Y.S., Y.Z., B.L. and A.F. contributed to analysis and interpretation of data. D.W., X.Z., T.Y. and T.F. assisted with data analysis. All authors contributed to interpretation of data and study findings. Y.S., T.Y., T.F. and H.Z. drafted the manuscript, with all authors critically reviewing the paper. All authors have read and approved the final report.

## FUNDING

This study was supported by the Natural Science Foundation of China Excellent Young Scientists Fund (82022064), Natural Science Foundation of China International/Regional Research Collaboration Project (72061137001), Natural Science Foundation of China Young Scientist Fund (81703278), the National Science and Technology Major Project of China (2018ZX10721102), the Sanming Project of Medicine in Shenzhen (SZSM201811071), the High Level Project of Medicine in Longhua, Shenzhen (HLPM201907020105), the National Key Research and Development Program of China (2020YFC0840900), the Shenzhen Science and Technology Innovation Commission Basic Research Program (JCYJ20190807155409373), Special Support Plan for High‐Level Talents of Guangdong Province (2019TQ05Y230), the Fundamental Research Funds for the Central Universities (58000‐31620005) and Non‐profit Central Research Institute Fund of Chinese Academy of Medical Sciences (2020).

Disclaimer: All funding parties did not have any role in the design of the study or in the explanation of the data.

## Supporting information



Additional file 2
Figure S1. Prespecified themes of barriers to ART maintenance and solutions to ART interruption. ART, antiretroviral therapy; CBO, community‐based organizations; CDC, centres for disease control and prevention; PLHIV, people living with HIV.

Supporting Materials
