# Supplementary material for: Stakeholder efforts to mitigate antiretroviral therapy interruption among people living with HIV during the COVID‐19 pandemic in China: a qualitative study
Source: J Int AIDS Soc. 2021 Sep 2;24(9):e25781. doi: 10.1002/jia2.25781 (PMC8412021; doi:10.1002/jia2.25781)
Supplement: Supplementary file 1 — Additional file 2 Figure S1. Prespecified themes of barriers to ART maintenance and solutions to ART interruption. ART, antiretroviral therapy; CBO, community‐based organizations; CDC, centres for disease control and prevention; PLHIV, people living with HIV. [file JIA2-24-e25781-s002.docx]

**Study setting**

**Voice call interview:** One investigator will call the participant in a quiet environment to ensure the quality of audio-record. The interview will be conducted according to the semi-structured interview guides, and will ask opened-ended follow-up questions after closed-ended questions to ensure people living with HIV (PLHIV) and stakeholders had an opportunity to expand on their earlier responses and share relevant experiences not directly prompted through earlier questions.

**Text message conversation:** Similar procedure as voice call interview, change the conversation way from voice to text.

**Sample size of interest**

Previous similar research found that saturation of themes was reached after 12 interviews on average (1). Hence, we planned to interview 15 participants from each group of PLHIV, community-based organization (CBO) workers, Centers for Disease Control and Prevention (CDC) staff, HIV doctors and nurses. For drug vender group, we aimed to recruit two representatives as supplement. We would adjust those recruit number depending on when data saturation was achieved.

**The role of ART in population of interest**

**PLHIV:** need to take ART.

**CBO worker:** provide health education and counselling, some of them have free ART reserve.

**CDC staff:** be responsible for ART delivery or ART management.

**HIV doctors and nurses:** be responsible for ART delivery.

**Drug vendor:** sell PLHIV the out-of-pocket ART.

**Reference:**

1. Vasileiou K, Barnett J, Thorpe S, Young T. Characterising and justifying sample size sufficiency in interview-based studies: systematic analysis of qualitative health research over a 15-year period. BMC medical research methodology. 2018;18(1):148.

**Supplemental Table S1. Interview questions for PLHIV**

| **ID** | **Questions** |
| --- | --- |
| 1 | When did you diagnose HIV? What is the CD4 cell count at diagnosis? When is the most recent time for your testing CD4 cell counts and what was the result? When did you initiate ART? What is your current regimen option? Where and how did you often obtain it? Did you get it through designated clinic or by mailing? |
| 2 | Have you ever been at risk of ART interruption before the COVID-19 pandemic? Why? How did you deal with it? |
| 3 | During the COVID-19 pandemic, how many drugs did you had when you were trying dealing with the ART interruption? What measures did you take to get ART refilling? What difficulties did you meet? Did you try to refill ART by mail or express? If yes, by which courier company? What’s the biggest problems did you encounter during the mailing process? |
| 4 | During the COVID-19 pandemic, did you encounter the situation where the CDC clinics or hospitals did not store your ART regimen? Did you turn to other solutions? Have you ever consider changing your regimen option or switching it to out-of-pocket ART? |
| 5 | During the COVID-19 pandemic, could you refill ART under traffic control? Under this situation, what measures did you take to obtain ART and what difficulties did you encounter? |
| 6 | During the COVID-19 pandemic, were you afraid of privacy disclosure when you were obtaining or borrowing ART? Did it actually happen? Did it have an impact on you? |
| 7 | During the COVID-19 pandemic, did you worry about contracting COVID-19? Did you think you have suspected symptoms? Any other mental problems? If you had, have you solve it and how? |
| 8 | Do you know the directive of “Notice on Ensuring Free Antiviral Therapy Drugs for Stranded People Living with HIV”? When did you hear about it? What’s your opinions about the measures taken by the government and the health department? Were there any problems? |
| 9 | Do you have any further comments or suggestions for the government and health departments to solve the problem of ART interruption in the future? |

ART, antiretroviral therapy; CDC, Centers for Disease Control and Prevention; PLHIV, people living with HIV.

**Supplemental Table S2. Interview questions for CBO worker**

| **ID** | **Questions** |
| --- | --- |
| 1 | Were your working hours affected since the COVID-19 pandemic? If yes, during COVID-19, how long do you work per day on average and when does this state start? Did you take a break during this period? How many days did you rest? How long did you work per day before the COVID-19 pandemic? |
| 2 | Did your organization remind PLHIV to getting some ART reserve during the Spring Festival previously? How about this year? |
| 3 | When did you receive the first PLHIV requests since the COVID-19? How many people have asked for help so far? Through which ways? What were the main problems they encountered? What kind of support did your organization offer? |
| 4 | Do you know the directive of “Notice on Ensuring Free Antiviral Therapy Drugs for Stranded People Living with HIV”? When did you first hear about it? Is there any difference in the number of consultants and help-seeking information of PLHIV before and after the document issued? |
| 5 | How many doses of drugs did PLHIV usually borrow? How many of them were at risk of ART interruption (less than 10 days of ART reserve)? How many doses of drugs did you usually borrow for them? Have you cooperated with other CBOs? |
| 6 | For PLHIV who cannot obtain ART due to traffic control, what measures have you taken? Compared with local PLHIV, what were the difficulties for PLHIV who stranded in your city or outside applying drugs from you? How did you deal with this situation? Do you provide services of ART mailing? Which courier companies do you usually choose? What difficulties did you encounter during the mailing process? |
| 7 | How did you coordinate with ART borrowing, and how did it work? What difficulties did you encounter when helping PLHIV consulting with designated CDC clinics and hospitals? How did you deal with it? |
| 8 | During the COVID-19 pandemic, did you provide mental health counselling to PLHIV? By what ways? What were the main problems they faced? |
| 9 | During the COVID-19 pandemic, what are the confidentiality requirements of PLHIV when they borrowed ART? What’s your opinion about it and how did you deal with it? |
| 10 | The pandemic is still ongoing, and traffic control is still strict. More and more PLHIV may face the problem of ART interruption. What is your next plan? Are there any ART refilling services available online in your organization? Was it offered in the past? What are the special arrangements during the pandemic? In your opinion, what would be the gap to facilitate ART delivery? |
| 11 | Do you have any comments or suggestions for the government and health departments to solve the problem of ART interruption in the future? |

ART, antiretroviral therapy; CBOs, community-based organizations; CDC, Centers for Disease Control and Prevention; PLHIV, people living with HIV.

**Supplemental Table S3. Interview questions for CDC staff**

| **ID** | **Questions** |
| --- | --- |
|  | - **Responsible for ART delivery: (Yes-Version one, No- Version two)** |
|  | **(Version one)** |
| 1 | During the COVID-19 pandemic, were you assigned with additional task for COVID-19 prevention, addressing double pressure from your work? How long do you work per day on average? When did this state start? Did you take a break during this period? How many days did you rest? How long did you work per day before the COVID-19 pandemic? |
| 2 | How many PLHIV are currently on ART in your organization? How many are registered locally? How many PLHIV could obtain enough drugs for next month? How many drugs have been sent to PLHIV stranded in other places? The pandemic is spreading and PLHIV may face new problems over time. How long can the current ART reserve be available? |
| 3 | During the COVID-19 pandemic, how many PLHIV obtained ART from your department per day on average? How many were stranded in your city? How many drugs did they generally apply for? How many of them were at risk of ART interruption (less than 10 days of drug reserve)? What are the reasons of their ART interruption? How many doses of drugs do you give them? How many were given in the past? Did you encounter temporary ART borrowing in the past? How did you deal with it? |
| 4 | During the COVID-19 pandemic, have you encounter any suspected or confirmed cases of COVID-19 among the PLHIV? Are their ART prescription adjusted after infection? |
| 5 | What is the reason for PLHIV who stranded in your city with the incomplete formalities to refill ART? Would you provide ART first and then asked for supplement of formalities later? In addition, why did PLHIV failed to obtain ART? |
| 6 | For PLHIV who cannot obtain ART due to traffic control, what measures have you taken? Compared with local PLHIV, what were the difficulties for PLHIV who stranded in your city or outside applying drugs from you? How did you deal with these? Do you provide services of ART mailing? Which courier companies do you usually choose? What difficulties did you encounter during the mailing process? |
| 7 | During the COVID-19 pandemic, did you provide mental health counselling to PLHIV? By which ways? What are the main problems they faced? |
| 8 | During the COVID-19 pandemic, what are the confidentiality requirements of PLHIV when they borrow ART? What’s your opinion about it and how did you deal with it? |
| 9 | The pandemic is still ongoing, and traffic control is still strict. More and more PLHIV may face the problem of ART interruption. What is your next plan? Did you purposively contact PLHIV who might at risk of ART interruption? Are there any ART refilling services available online in your organization? Was it offered before? What are the special arrangements during the pandemic? In your opinion, what would be the gap that make progress to achieve ART delivery more reasonable? |
| 10 | Do you have any comments or suggestions for the government and health departments to solve the problem of ART interruption in the future? |
|  | **(Version two)** |
| 1 | During the COVID-19 pandemic, were you assigned with additional task for COVID-19 prevention, addressing double pressure from your work? How long do you work per day on average? When did this state start? Did you take a break during this period? How many days did you rest? How long did you work per day before the COVID-19 pandemic? |
| 2 | Which kind of work related to ART delivery you are responsible for? How many PLHIV are currently on ART in your organization? How many PLHIV are registered locally? |
| 3 | During the COVID-19 pandemic, what problems did you encounter when you were planning ART reserve, communicating and coordinating with the ART-distributing organizations? Have you encountered this before the COVID-19 pandemic? |
| 4 | The pandemic is still ongoing, and traffic control is still strict. More and more PLHIV may face the problem of ART interruption. What is your next plan? Did you purposively contact PLHIV who might be at risk of ART interruption? Are there any ART refilling services available online in your organization? Was it offered before? What are the special arrangements during the pandemic? In your opinion, what would be the gap that make progress to achieve ART delivery more reasonable? |
| 5 | Do you have any comment or suggestion for the government and health departments to solve the problem of ART interruption in the future? |

ART, antiretroviral therapy; CDC, Centers for Disease Control and Prevention; PLHIV, people living with HIV.

**Supplemental Table S4. Interview questions for HIV doctors and nurses**

| **ID** | **Questions** |
| --- | --- |
| 1 | During the COVID-19 pandemic, were you assigned with additional task for COVID-19 prevention, addressing double pressure from your work? How long do you work per day on average? When did this state start? Did you take a break during this period? How many days did you rest? How long did you work per day before the COVID-19 pandemic? |
| 2 | How many PLHIV are currently on ART in your organization? How many are registered locally? How many PLHIV could obtain enough drugs for next month? How many drugs have been sent to PLHIV stranded in other places? The pandemic is spreading and PLHIV may face new problems over time. How long can the current ART reserve be available? |
| 3 | During the COVID-19 pandemic, how many PLHIV obtained ART from you per day on average? How many were stranded locally? How many drugs did they generally apply for? How many of them were at risk of ART interruption (less than 10 days of drug reserve)? What are the reasons of their ART interruption? How many doses of drugs do you give them? How many were given in the past? Have you ever encountered temporary ART borrowing in the past? How did you deal with it? |
| 4 | Is the HIV clinic in your hospital closed? If, yes, when did it close? Are there any suspected or confirmed cases of COVID-19 among PLHIV you are responsible for? Are their ART prescription adjusted after infection? |
| 5 | What is the reason for PLHIV who stranded in your city with the incomplete formalities to refill ART? Would you provide ART first and then asked supplement of the formalities later? In addition, what are the reasons why PLHIV failed to obtain ART? |
| 6 | For PLHIV who cannot obtain ART due to traffic control, what measures have you taken? Compared with local PLHIV, what were the difficulties for PLHIV who stranded in your city or outside applying drugs from you? How did you deal with these? Do you provide services of ART mailing? Which courier companies do you usually choose? What difficulties did you encounter during the mailing process? |
| 7 | During the COVID-19 pandemic, did you provide mental health counselling to PLHIV? By which ways? What are the main problems they faced? |
| 8 | During the COVID-19 pandemic, what are the confidentiality requirements of PLHIV when they borrowed ART? What’s your opinion about it and how did you deal with it? |
| 9 | The pandemic is still ongoing, and traffic control is still strict. More and more PLHIV may face the problem of ART interruption. What is your next plan? Did you purposively contact PLHIV who might at risk of ART interruption? Are there any ART refilling services available online in your organization? Was it offered before? What are the special arrangements during the pandemic? In your opinion, what would be the gap that make progress to achieve ART delivery more reasonable? |
| 10 | Do you have any comment or suggestion for the government and health departments to solve the problem of ART interruption in the future? |

ART, antiretroviral therapy; PLHIV, people living with HIV.

**Supplemental Table S5. Interview questions for drug vendor**

| **ID** | **Questions** |
| --- | --- |
| 1 | During the COVID-19 pandemic, how long do you spend on ART sales and consultation per day? When did this state start? Have you ever taken a break during this period? How many days did you rest? How long did you work per day before the COVID-19 pandemic? |
| 2 | What kind of ART have you sold most? How many PLHIV would buy ART every month on average before the COVID-19 pandemic, and how much doses would they buy at a time? Are there any changes in the types and count of ART they wanted during and before the COVID-19 pandemic? |
| 3 | When did PLHIV’s demand for ART increase during the COVID-19 pandemic? How many PLHIV have consulted about ART so far? Did they ask help through Weibo or WeChat? Any other ways if available? How many PLHIV changed their ART prescription from free to out-of-pocket ones, or took out-of-pocket ART as temporary substitution? Did they buy your drugs after consultation, if not, Why? |
| 4 | Which way did you mainly obtain ART before the COVID-19 pandemic? Are there any new ways during the COVID-19 pandemic? What about the difference of supplement among different ways? What difficulties did you encounter in the process of ART reserve? |
| 5 | During the COVID-19 pandemic, did your regular customers request for more drugs? How many doses did they buy at a time? What about new customers? How many of your customers were at risk of ART interruption (less than 10 days of ART reserve)? Were you able to meet their purchase requirement? Have any customers consulted about purchasing national free ART? Could you provide? By selling, donating, or just refusing? Did you encounter other difficulties in communicating with customers? How did you deal with it? |
| 6 | Did you offer mailing service? Which courier companies do you usually choose? How many PLHIV failed to obtain ART via mailing due to suspension of courier services? What else difficulties did you encounter during the mailing process? |
| 7 | During the COVID-19 pandemic, what are the confidentiality requirements of PLHIV when they bought ART? What’s your opinion about it and how did you deal with it? |

ART, antiretroviral therapy; PLHIV, people living with HIV.
