# Supplementary figures and images for: Stakeholder efforts to mitigate antiretroviral therapy interruption among people living with HIV during the COVID‐19 pandemic in China: a qualitative study
Source: J Int AIDS Soc. 2021 Sep 2;24(9):e25781. doi: 10.1002/jia2.25781 (PMC8412021; doi:10.1002/jia2.25781)

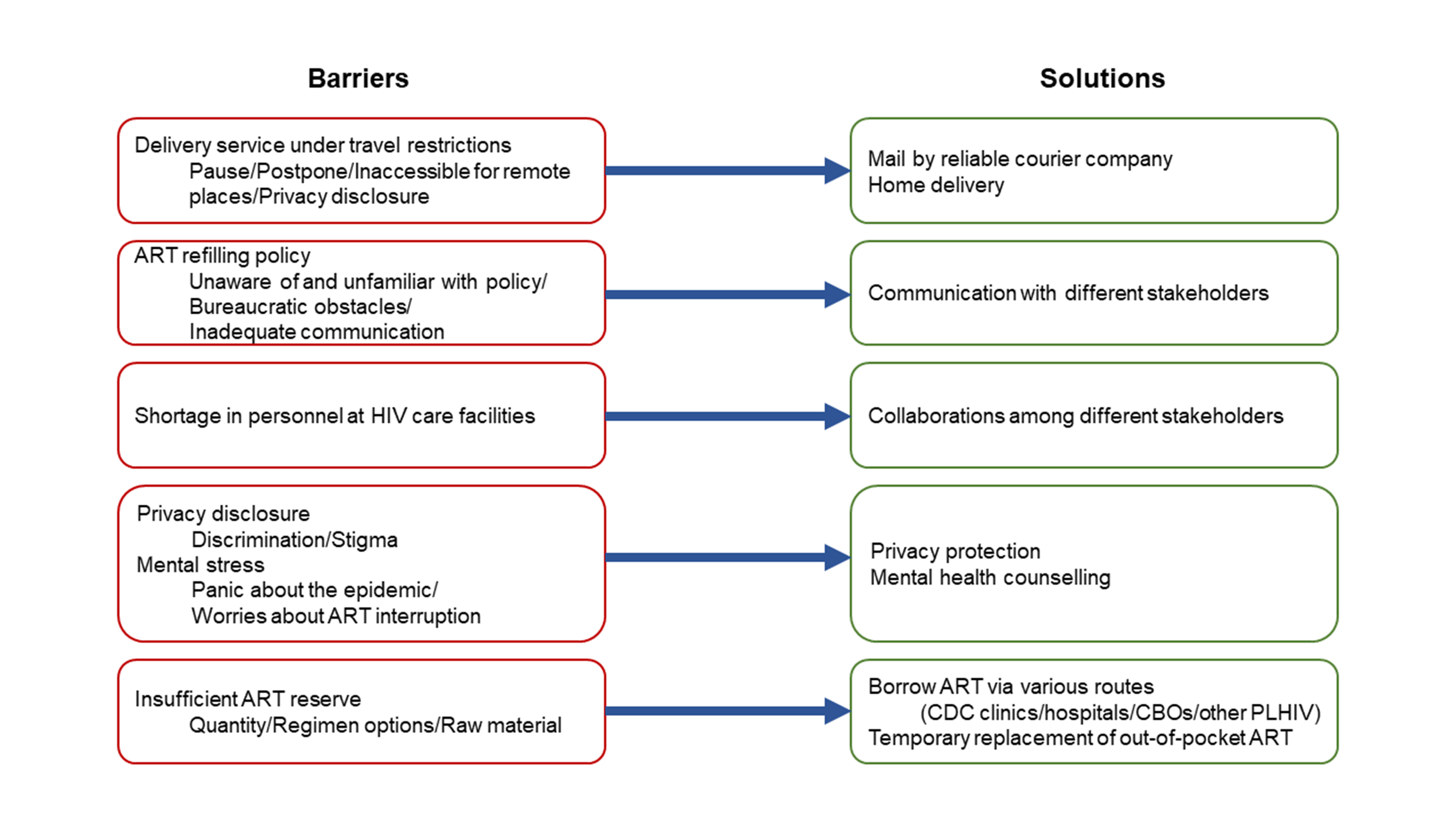

Supplement: Supplementary file 2 — Supporting Materials [file JIA2-24-e25781-s001.tif]
